# Is biofeedback-assisted pelvic floor muscle training superior to pelvic floor muscle training alone in the treatment of dysfunctional voiding in women? A prospective randomized study

**DOI:** 10.1590/S1677-5538.IBJU.2021.0687

**Published:** 2022-03-14

**Authors:** Emre Sam, Ahmet Emre Cinislioglu, Fatih Kursat Yilmazel, Saban Oguz Demirdogen, Ali Haydar Yilmaz, Ibrahim Karabulut

**Affiliations:** 1 University of Health Sciences Regional Training and Research Hospital Department of Urology Erzurum Turkey Department of Urology, University of Health Sciences, Regional Training and Research Hospital, Erzurum, Turkey; 2 Bilecik State Hospital Department of Urology Bilecik Turkey Department of Urology, Bilecik State Hospital, Bilecik, Turkey

**Keywords:** Pelvic Floor, Women, Lower Urinary Tract Symptoms

## Abstract

**Purpose:**

To compare the effectiveness of biofeedback-assisted pelvic floor muscle training (PFMT) and PFMT alone on voiding parameters in women with dysfunctional voiding (DV).

**Materials and Methods:**

The patients in group 1 (34 patients) were treated with biofeedback-assisted PFMT, and the patients in group 2 (34 patients) were treated with PFMT alone for 12 weeks. The 24-hour frequency, average voided volume, maximum urine flow rate (Q_max_), average urine flow rate (Q_ave_), post-void residual urine volume (PVR), and the validated Turkish Urogenital Distress Inventory (UDI-6) symptom scores were recorded before and after 12 weeks of treatment.

**Results:**

At the end of treatment sessions, the Q_max_ and Q_ave_ values of the patients in group 1 were significantly higher than those in group 2, and the PVR in the patients in group 1 was significantly lower than those in group 2 (p=.026, .043, and .023, respectively). The average UDI-6 symptom scores of the patients in group 1 were significantly lower than those in group 2 (p=.034). Electromyography activity during voiding, in group 1 was significantly lower than in group 2 (41.2 vs. 64.7, respectively, p=.009).

**Conclusion:**

Biofeedback-assisted PFMT is more effective than PFMT alone in improving clinical symptoms, uroflowmetry parameters, and EMG activity during voiding.

## INTRODUCTION

Voiding dysfunction refers to abnormally slow, intermittent voiding and/or incomplete bladder emptying (1). There are two main types of voiding dysfunction: bladder outlet obstruction (BOO) and detrusor underactivity (DU). Functional disorders or structural lesions (mechanical obstruction) may cause the development of BOO. Disorders such as detrusor sphincter dyssynergia and dysfunctional voiding (DV) are among the causes of functional BOO (2). The International Society of Continence defines DV as an intermittent and/or fluctuating flow rate due to involuntary intermittent contractions of the periurethral striated or levator muscles during voiding in neurologically normal individuals (1). Simultaneous contraction of the sphincter and detrusor in the presence of a neurological condition is defined as detrusor sphincter dyssynergia (3, 4). The lack of coordination between the detrusor and sphincter is similar in both disorders; however, the etiologies are different, and these two terms cannot be used synonymously (5).

A variety of lower urinary tract symptoms (LUTS) can occur in DV, including storage symptoms (frequency, stress, and urge incontinence) and emptying symptoms (poor or intermittent stream, hesitancy, and feeling of incomplete emptying). It may cause recurrent urinary tract infections and acute or chronic urinary retention (2, 6). The goal of treatment is to normalize the voiding patterns and prevent complications. Despite various treatments that have been tried, there is, unfortunately, no treatment modality that can be recommended with high levels of evidence for DV due to limited data (7).

The muscle groups targeted with pelvic floor muscle training (PFMT) are the levator ani, the external anal sphincter, and the striated urethral sphincter. These exercises aim to increase muscle tone and synchronize contractions (8). The ability to properly contract the pelvic floor muscles is essential for PFMT. Women who are able to contract their pelvic floor muscles correctly are suitable for PFMT (9). The goal of biofeedback is to increase awareness of the function of the pelvic floor muscles and to develop better voluntary control of these muscles and the external urethral sphincter during voiding. Biofeedback is not a therapy by itself but an adjunct to PFMT in measuring the response from the contraction of the pelvic floor muscles (10, 11).

We hypothesized that PFMT may be an effective treatment modality for women with DV, and also that combining PFMT with biofeedback may provide more effective pelvic floor muscle awareness and more successful outcomes than PFMT alone. Therefore, we aimed to compare the effectiveness of biofeedback-assisted PFMT and PFMT alone on the voiding parameters of patients by implementing an effective training program in women with DV.

## MATERIALS AND METHODS

This prospective randomized study was approved by the local ethics committee (Approval number: B.30.2.ATA.0.01.00/999).

### Participants

Women aged 18-50 years, who presented to our clinic with LUTS between May 2019 and August 2020, were evaluated. Uroflowmetry and post-void residual urine volume (PVR) by ultrasound were performed. Patients with a maximum urine flow rate (Q_max_) ≤ 15 mL/s and/or PVR > 50 mL in at least two measurements were diagnosed with voiding dysfunction (12). Patients with advanced pelvic organ prolapse (Stage III and IV), diabetes mellitus, neurological disorder (e.g., multiple sclerosis, spinal cord compression, Parkinson's disease, lumbar disc prolapse, or spina bifida), history of lower urinary system surgery or intra-abdominal radiotherapy, active urinary system infection, and urethral stricture/anatomic obstruction were excluded from the study. A 12 Fr Foley catheter was advanced in all patients to exclude urethral stricture or anatomic obstruction. Patients with difficulty advancing the Foley catheter were examined by ureterorenoscope. The remaining patients underwent uroflowmetry with electromyography (EMG). A total of 82 patients with simultaneous detrusor and external sphincter activity during voluntary voiding on EMG were diagnosed with DV. Eight patients refused to participate, and a total of 74 patients were included in the study. The sample size was determined based on the studies on DV in women.

**Figure 1:**
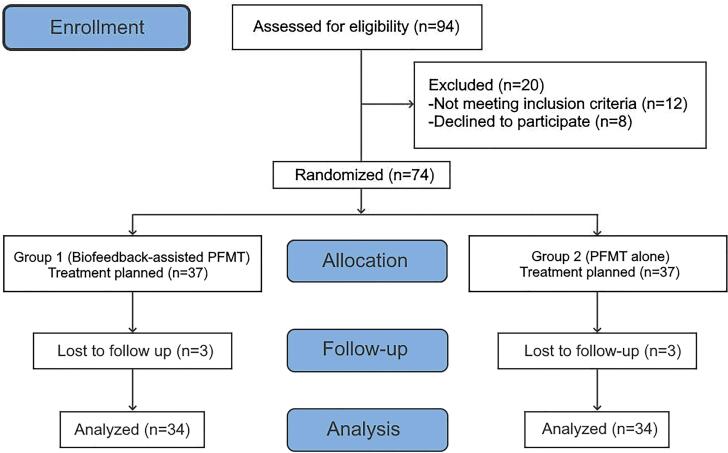
CONSORT flow diagram. The diagram illustrates the progress of the participants through the enrollment, allocation, follow-up, and analysis phases of the trial.

The patients’ ability to perform contraction and relaxation and their compliance with PFMT were evaluated with digital palpation by the urotherapist. Patients were randomly assigned using block randomization (a computer-generated list of random numbers) by the researcher (IK) and divided into two groups. The patients in group 1 were treated with biofeedback-assisted PFMT, and the patients in group 2 were treated with PFMT alone for 12 weeks. None of the patients performed PFMT before and had no knowledge of PFMT. The patients in both groups were trained in the clinic by the same PFMT certified urotherapist. To ensure compliance with the exercise program, the patients were asked to fill in the follow-up chart, and these charts were checked by the urotherapist every week. Comprehensive information was given to the patients, emphasizing the importance of regular and consistent exercise. Six patients were excluded from the study because they did not want to continue. The study was completed with a total of 68 patients: 34 in group 1 and 34 in group 2 (Figure-1).

Before and after the 12-week treatment, uroflowmetry (Q_max_; average urine flow rate: Q_ave_), bladder diary (24-hour frequency and average voided volume), PVR, the validated Turkish Urogenital Distress Inventory (UDI-6) (13), and EMG activity during voluntary voiding were evaluated in the patients in both groups.

The UDI-6 is a six-item scale developed to evaluate bladder function and the problem-causing symptoms. The first and second questions assess urgency, frequency, and pain; the third and fourth questions assess the stress symptoms; the fifth and sixth questions assess the obstructive/discomfort or symptoms of voiding difficulty.

### Primary and Secondary Objectives

The primary objective of our study was to determine whether the 12-week biofeedback-assisted PFMT or PFMT alone is an effective treatment for women with DV. Treatment efficacy was evaluated with the UDI-6 score. The secondary objective was to determine the effects of 12-week biofeedback-assisted PFMT or PFMT alone on bladder diary, uroflowmetry parameters, PVR, and EMG activity during voiding in the treatment of women with DV.

### Biofeedback-Assisted Pelvic Floor Muscle Training

The patients in group 1 were given the necessary anatomical information at the beginning of the biofeedback-assisted PFMT and taught the exercises with one-to-one supervision by a urotherapist. The patients were asked to empty their bladders before the procedure. They were positioned in the supine position with their knees slightly flexed and their heads slightly raised. Surface EMG probes were placed in the three and nine o'clock positions on the perineum, an additional neutral probe was placed on the patella, and the patients were monitored. The patients were asked only to contract their pelvic floor muscles, not their abdominal muscles. They were also asked to follow the contraction and relaxation of their pelvic floor muscles on a monitor and to make sure that they were contracting the correct muscle group; thus, enabling active participation in the education program. By this means, the patients were taught how to identify their pelvic floor muscles and how to use their pelvic floor muscles selectively without using their abdominal muscles. The patients were enrolled in biofeedback-assisted PFMT sessions three times a week (a total of 60 minutes, with each session lasting an average of 20 minutes) for 12 weeks. Moreover, the patients were given an unsupervised standard PFMT program for home practice in which the intensity of 12-week exercise increased gradually and systematically (Supplementary Material – Appendix 1).

### Pelvic Floor Muscle Training Alone

The patients in group 2 were given the necessary anatomical information at the beginning of the PFMT and taught the exercises with one-to-one supervision by a urotherapist. The patients were asked to empty their bladders before the procedure. They were asked to lie in the supine position, insert a finger into the vagina, and contract and relax the pelvic floor muscles (not their abdominal muscles), noticing the contraction around the finger. Furthermore, the patients were given an unsupervised standard PFMT program for home practice in which the intensity of 12-week exercise increased gradually and systematically (Supplementary Material – Appendix 1).

### Uroflowmetry/EMG

**Figure 2:**
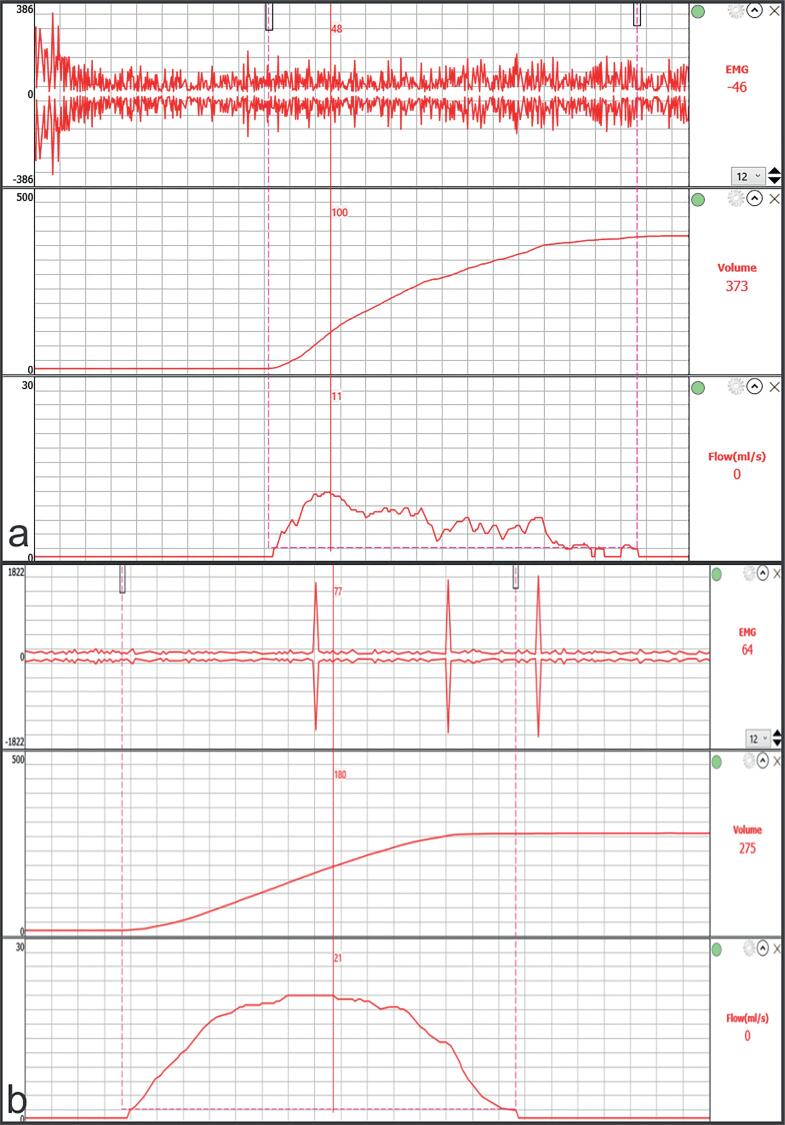
Electromyography + Uroflowmetry image of the same patient (a) before treatment and (b) at the end of treatment sessions.

All patients were evaluated with physiologically full bladders. Protocols were carried out by the same urologist using the Itri Pro system (Aymed Medical Technology, Istanbul, Turkey). EMG probes were placed in the three and nine o'clock positions in the perianal region, the neutral electrode was placed on the patella, and the patients were asked to urinate. During voluntary voiding, the urinary stream pattern and EMG activity were recorded. After the procedure, the PVR volume was measured by ultrasound. After the treatment, the protocol was repeated for both groups (Figure-2).

#### Statistical Analysis

The summary statistics of the variables were presented as means and standard deviations. The normality of the parameters was assessed with the D’Agostino Pearson test. Continuous variables in paired groups were compared using the paired t-test, whereas continuous variables belonging to different groups were compared using the Student's t-test. A two-tailed p-value of <0.05 was accepted as statistically significant. All statistical evaluations were performed using the R statistical software package (R Studio, Vienna, Austria).

## RESULTS

A diagnosis of DV was made in 9.8% of women who presented to the outpatient clinic with LUTS. The mean age of the patients in group 1 was 46.5 ± 9.9 years, and their body mass indexes were calculated as 24.8 ± 2.2 kg/m^2^. The mean age of the patients in group 2 was 43.1 ± 7.2 years, and their body mass indexes were calculated as 24.5 ± 2.2 kg/m^2^. Before treatment, there was no significant difference between patients in group 1 and group 2 in terms of age (p = 0.708), body mass index (p = 0.896), 24-hour frequency, average voided volume, Q_max_, Q_ave_, PVR, average UDI-6 symptom score, and EMG activity during voluntary voiding (Table-1).

**Table 1:** Comparison of groups with each other before treatment.

	Group 1	Group 2	p
	(n=34)	(n=34)	
Age, years (Mean ± SD)	46.5 ± 9.9	43.1 ± 7.2	0.708†
BMI, kg/m^2^ (Mean ± SD)	24.8 ± 2.2	24.5 ± 2.2	0.896†
24-hour frequency (Mean ± SD)	11.2 ± 2.1	11.2 ± 2.0	0.830
Average voided volume, mL (Mean ± SD)	252.2 ± 81.7	261.4 ± 94.4	0.668
Q_max_, mL/s, (Mean ± SD)	11.0 ± 2.7	11.1 ± 2.6	0.964
Q_ave_, mL/s (Mean ± SD)	6.0 ± 1.1	6.0 ± 1.1	0.918
PVR, mL (Mean ± SD)	83.2 ± 25.4	82.7 ± 24.8	0.942
UDI-6 score (Median)	13.5	13.5	0.995
EMG activity, n (%)	34 (100)	34 (100)	1††

**BMI** = Body mass index;**Q_max_**:maximum urine flow rate; **Q_ave_** = average urine flow rate; **PVR** = Post-void residual urine volume; **UDI-6** = Urogenital Distress Inventory; **EMG** = electromyography

† Kruskal Wallis

†† Intergroup comparison was performed using the Chi-square test

In the patients in group 1, there was no significant difference in the average voided volume and EMG activity during voluntary voiding before and after biofeedback-assisted PFMT, whereas Q_max_, Q_ave_, PVR, and UDI-6 symptom scores were significantly different. In the patients in group 2, there was no significant difference in the average voided volume and EMG activity during voluntary voiding before and after PFMT, whereas Q_max_, Q_ave_, PVR, and the average UDI-6 symptom scores were significantly different (Table-2).

**Table 2:** Intra-Group comparisons of voiding parameters before and after treatment.

	Group 1 (n=34)			Group 2 (n=34)		
	Before treatment	After treatment	p	Before treatment	After treatment	p
24-hour frequency (Mean ± SD)	11.2 ± 2.1	7.7 ± 2.9	**<0.001**	11.2 ± 2.0	9.0 ± 3.1	**0.008**
Average voided volume, mL (Mean ± SD)	252.2 ± 81.7	261.7 ± 77.5	0.622	261.4 ± 94.4	258.0 ± 82.0	0.815
Q_max_, mL/s, (Mean ± SD)	11.0 ± 2.7	14.8 ± 2.6	**0.001**	11.1 ± 2.6	12.8 ± 2.9	**0.005**
Q_ave_, mL/s (Mean ± SD)	6.0 ± 1.1	8.0 ± 1.0	**0.001**	6.0 ± 1.1	6.7 ± 1.1	**0.007**
PVR, mL (Mean ± SD)	83.2 ± 25.4	54.7 ± 31.5	**<0.001**	82.7 ± 24.8	65.7 ± 24.1	**0.005**
UDI-6 score (Median)	13.5	9	**<0.001**	13.5	12	**0.002**
EMG activity, n (%)	34 (100)	14 (41.2)	0.391†	34 (100)	22 (64.7)	0.122†

**Q_max_** = maximum urine flow rate; **Q_ave_** = average urine flow rate; **PVR** = Post-void residual urine volume; **UDI-6** = Urogenital Distress Inventory; **EMG** = electromyography

† Intergroup comparison was performed using the Chi-square test

At the end of treatment sessions, there was no significant difference between group 1 and group 2 in the 24-hour frequency and the average voided volume. The Q_max_ and Q_ave_ values of the patients in group 1 were significantly higher than those in group 2 (p = 0.026 and p = 0.043, respectively), and the PVR in the patients in group 1 was significantly lower compared to the patients in group 2 (p = 0.023). Furthermore, the average UDI-6 symptom scores of the patients in group 1 were significantly lower than those in group 2 (p = 0.034). During voluntary voiding, EMG activity continued in 64.7% of the patients in group 2, while this proportion was 41.2% in group 1 (p = 0.009) (Table-3).

**Table 3:** Inter-Group comparison of voiding parameters after treatment.

	Group 1 (n=34)	Group 2 (n=34)	p
24-hour frequency (Mean ± SD)	7.7 ± 2.9	9.0 ± 3.1	0.078
Average voided volume, mL (Mean ± SD)	261.7 ± 77.5	258.0 ± 82.0	0.850
Q_max_, mL/s, (Mean ± SD)	14.8 ± 2.6	12.8 ± 2.9	**0.026**
Q_ave_, mL/s (Mean ± SD)	8.0 ± 1.0	6.7 ± 1.1	**0.043**
PVR, mL (Mean ± SD)	54.7 ± 31.5	65.7 ± 24.1	**0.023**
UDI-6 score (Median)	9	12	**0.034**
EMG activity, n (%)	14 (41.2)	22 (64.7)	**0.009** †

**Q_max_** = maximum urine flow rate; **Q_ave_** = average urine flow rate; **PVR** = Post-void residual urine volume; **UDI-6** = Urogenital Distress Inventory; **EMG** = electromyography

† Intergroup comparison was performed using the Chi-square test

## DISCUSSION

The majority of DV studies have been conducted in the pediatric population, and the number of studies on women with DV is limited (14). Carlson et al. examined 134 patients who presented with LUTS using video-urodynamics and detected DV in 12% of the patients (15). Furthermore, Nitti et al. found DV to be the cause in 25 (33%) of 76 patients diagnosed with BOO by video-urodynamics (16). We also diagnosed DV in 9.8% of the women who presented to the outpatient clinic with LUTS. These findings show that a significant proportion of the women who present with LUTS have DV. However, in women, DV is likely to be underestimated, and this could lead to the diagnosis of DV being overlooked, thereby causing significant deterioration in the quality of life of women with DV.

It is difficult to determine whether voiding dysfunction is caused by BOO or DU only by looking at the symptoms (17). Therefore, urodynamics is required; however, there is no consensus on urodynamic parameters that provide the most accurate diagnosis of BOO in women. Chassagne et al. reported that it would be reasonable to diagnose BOO in women using the pressure-flow study (Q_max_≤15 mL/s and pdet. Q_max_>20 cmH_2_O with 74.3% sensitivity and 91.1% specificity) (18). Nitti et al. suggested that BOO in women should be diagnosed radiologically with video-urodynamics. They determined Q_max_ as >15 mL/s in 11.8% and pdet. Q_max_ as <20 cmH_2_O in 10.5% of the patients with an obstruction in the radiologic examination, and suggested that pressure-flow studies alone may fail to diagnose obstruction (16). Blaivas and Groutz defined a nomogram for the diagnosis of BOO in women by combining uroflowmetry, pressure-flow study, and voiding cystourethrography (19).

The two most useful screening tests for detecting voiding difficulties, or abnormally slow or incomplete voiding are uroflowmetry and PVR measurement. Constantini et al. found that uroflowmetry had a high specificity (>70%) and negative predictive value (>79%) for the diagnosis of voiding dysfunction and argued that it was beneficial as the first diagnostic test to exclude voiding difficulty (20). These two tests are both indicative; however, they are not sufficient for the diagnosis of DV and should be combined with EMG (5, 21). The International Children's Continence Society provided a consensus document on the diagnosis of DV by uroflowmetry with EMG or video-urodynamics and declared that the literature on the necessity of invasive studies such as voiding cystourethrography and full urodynamic studies to diagnose DV in children is limited, and there has been a trend toward relying on less invasive studies in recent years (22, 23).

In this study, we evaluated women presenting with LUTS first with uroflowmetry and PVR. We diagnosed voiding dysfunction in patients with abnormal uroflowmetry and/or PVR findings and confirmed the diagnosis of DV by evaluating the EMG activity during voiding in neurologically normal women without anatomical obstruction. Although invasive urodynamics generally gives valuable information in women, its contribution to the diagnosis and management of BOO is not as clear as in men. Therefore, there may be no need to apply invasive urodynamics at first to patients who have not received any therapy yet and will be scheduled for a conservative non-invasive treatment without side effects, such as PFMT. The findings of this study also support our opinion. We believe that our diagnostic algorithm is the most practical, non-invasive, and cost-effective method.

The literature on DV treatment in women is limited. In a prospective randomized study conducted by Minardi et al., biofeedback-assisted PFMT was determined to improve the storage and emptying symptoms, objective urodynamic parameters, PVR, and the incidence of urinary tract infection significantly compared to the control group (24). In a prospective cohort study conducted by Chiang et al., biofeedback-assisted PFMT was effective in more than 80% of 31 women with DV, with significant improvements in clinical symptoms, QoL, and uroflowmetry parameters (25). To the best of our knowledge, this study is the first prospective randomized study comparing biofeedback-assisted PFMT and PFMT alone in women diagnosed with DV. We found that biofeedback-assisted PFMT was superior to PFMT alone in clinical symptoms evaluated with the UDI-6 score, uroflowmetry parameters, PVR, and EMG activity during voiding. This may be due to patients gaining pelvic floor muscle awareness more easily, thanks to biofeedback. Besides, the success of PFMT depends on patients correctly understanding the given tasks and their compatibility with the program. Biofeedback-assisted PFMT can minimize patient-related failures through sessions under the supervision of a urotherapist. Therefore, it would be a more rational approach to recommend biofeedback-assisted PFMT to women with DV.

Our study has some limitations. The fact that there is no consensus on the definitive diagnostic criteria of DV is a significant limitation of the study. Another important limitation is the fact that the patients did not perform invasive urodynamics or video-urodynamics. A clearer differential diagnosis between BOO and DU with invasive urodynamics or video-urodynamics and a comparison of pre- and post-treatment pressure-flow data would have made the study more valuable. Other limitations are the small number of patients and the shortness of the follow-up period.

## CONCLUSION

PFMT is a preferable treatment method due to its efficiency in DV management, easy applicability, and absence of side effects. Biofeedback-assisted PFMT is more effective than PFMT alone in improving clinical symptoms, uroflowmetry parameters, and EMG activity during voiding. Large comprehensive prospective randomized studies on this subject are necessary.

## ABBREVIATIONS

BOO: bladder outlet obstruction
DU: detrusor underactivity
DV: dysfunctional voiding
LUTS: lower urinary tract symptoms
PFMT: pelvic floor muscle training
PVR: post-void residual urine volume
Q_max_: maximum urine flow rate
EMG: electromyography
Q_ave_: average urine flow rate
UDI-6: the validated Turkish Urogenital Distress Inventory

## Appendix 1 Supplementary Material

### 12 week-Pelvic Floor Muscle Training Protocol

**Faucet exercise (F):** Repeatedly and strongly contract and release your pelvic floor (like closing-opening a faucet)

**Elevator exercise (E):** Slowly contract the pelvic floor by counting 5- hold by counting 5- release by counting 5 (like the elevator ascending by counting 5- holding at the top floor by counting 5- descending by counting 5)

**Table:**

Weeks	Exercise intensity per set	Number of sets per day	Total exercise intensity per day
1st and 2nd week	10 F + 10 E	5 sets	50 F + 50 E
3rd and 4th week	12 F + 12 E	5 sets	60 F + 60 E
5th and 6th week	15 F + 15 E	5 sets	75 F + 75 E
7th and 8th week	20 F + 20 E	5 sets	100 F + 100 E
9th and 10th week	25 F + 25 E	5 sets	125 F + 125 E
11th and 12th week	30 F + 30 E	5 sets	150 F + 150 E

**P.S** = One set was skipped on the days when biofeedback was applied.
